# Increasing protein stability by inferring substitution effects from high-throughput experiments

**DOI:** 10.1016/j.crmeth.2022.100333

**Published:** 2022-11-14

**Authors:** Rasmus Krogh Norrild, Kristoffer Enøe Johansson, Charlotte O’Shea, Jens Preben Morth, Kresten Lindorff-Larsen, Jakob Rahr Winther

**Affiliations:** 1 Linderstrøm-Lang Centre for Protein Science, Department of Biology, University of Copenhagen, 2200 Copenhagen N, Denmark; 2 Department of Biotechnology and Biomedicine, Technical University of Denmark, 2800 Kgs. Lyngby, Denmark

**Keywords:** protein design, deep mutational scanning, functional screening, GMMA, protein stability

## Abstract

We apply a computational model, global multi-mutant analysis (GMMA), to inform on effects of most amino acid substitutions from a randomly mutated gene library. Using a high mutation frequency, the method can determine mutations that increase the stability of even very stable proteins for which conventional selection systems have reached their limit. As a demonstration of this, we screened a mutant library of a highly stable and computationally redesigned model protein using an *in vivo* genetic sensor for folding and assigned a stability effect to 374 of 912 possible single amino acid substitutions. Combining the top 9 substitutions increased the unfolding energy 47 to 69 kJ/mol in a single engineering step. Crystal structures of stabilized variants showed small perturbations in helices 1 and 2, which rendered them closer in structure to the redesign template. This case study illustrates the capability of the method, which is applicable to any screen for protein function.

## Highlights

• Highly mutated protein libraries are rich in information when screened for function
• A global multi-mutant analysis (GMMA) can extract single substitution effects
• One-step engineering increased stability by ∼45% of already stable model protein
• GMMA is broadly applicable and complements structural and evolutionary models

## Motivation

Protein stability is an important parameter in almost all protein-engineering efforts. Evaluating the effects of the many possible amino acid changes to guide such projects is a significant task, even with recent advances in experimental and computational approaches. Thus, a generally applicable method to determine the effect on folding and stability of individual substitutions and in particular pinpointing multiple enhancing ones is missing. The work described here aims to devise such a method that should also be broadly applicable.

## Graphical abstract

**Figure:**
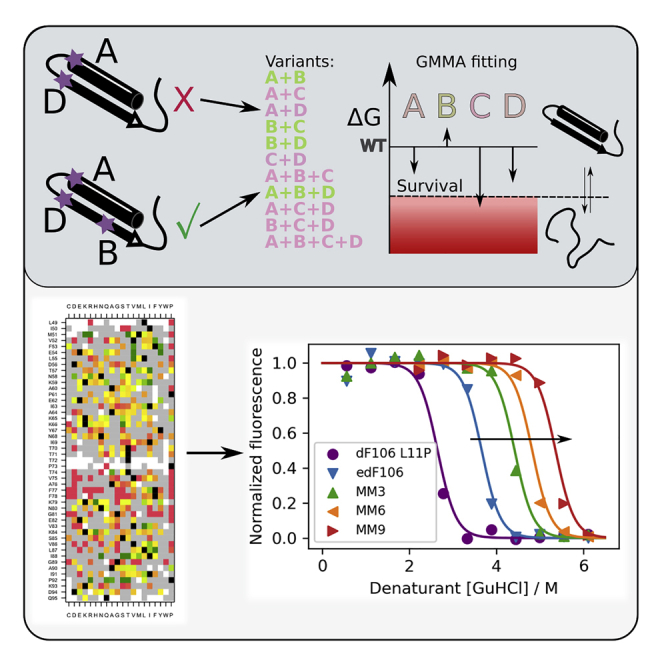

## Teaser

Thermodynamic stability is a key goal in protein engineering. Norrild et al. demonstrate how profiling of highly mutated protein variants combined with computational approaches to infer effects of single amino acid substitutions presents a richly informative approach to further engineer an already stable model protein, now melting at >150°C.

## Introduction

Protein engineering requires a complex concurrent optimization of function, stability, and other desired traits, which can be cumbersome even with efficient screening automation. One reason for this is that many substitutions may be required to reach the desired phenotype, and the combinatorial space, when introducing multiple substitutions in protein sequences, quickly rises to, and above, experimentally accessible numbers. Efficient ways to navigate this space are therefore highly desirable.^1^

Even in the cases where enhanced stability is not the primary goal, it may still be a useful starting point when engineering enzymes.^2^ As a general rule, proteins are only as stable as is required for the adequate fitness of their host.^3^ It therefore stands to reason that it should be possible to stabilize most mesophilic proteins and enzymes for biotechnology purposes. This can provide the necessary stability headroom to alter a protein’s function, for example, where modification of a substrate cavity is suboptimal for stability^4^ or when multiple destabilizing substitutions are required for directed evolution toward an altered function.^5^ Thus, increasing the stability of a protein can increase tolerance to substitutions,^6^ which might be needed for changes in the active site or other desired traits in protein engineering.

Directed evolution in combination with genetic selection and screening can address some of the challenges associated with the large sequence space. One of the tools that can be used to optimize stability is tripartite folding sensors.^7^ These are fusion proteins where the protein of interest (POI) is genetically inserted in a loop of a conditionally essential reporter enzyme.^8^ Given a stable POI, the reporter enzyme is catalytically active, and the organism survives, while an unstable POI renders the fusion protein misfolded, which abolishes the catalytic activity of the reporter, thus impeding growth. Mutants can be selected for increased stability of the POI by increasing temperature,^9^ antibiotic concentration,^10^ or by following fluorescent readout.^11^ Selection for aggregation resistance^12^ and identification of stable protein scaffolds^13^ can also achieved using such systems. They will, however, eventually reach the limit of their dynamic range, requiring more complicated approaches to increase stability further.^14^

Genetic screening systems, when combined with massively parallel sequencing (MPS), can be exploited for protein science in a range of powerful techniques broadly termed deep mutational scanning (DMS).^15^ The potential for applying the method for engineering has been shown in the optimization of a *de*-*novo*-designed influenza inhibitor^16^ and the identification of stabilizing substitutions by studying epistatic effects between the binding capabilities of single and double mutants.^17^ More quantitative analyses have been enabled by a thermodynamic model that considers doubly substituted protein variants to infer the effect of single amino acid substitutions on both protein-protein interaction and folding free energies,^18^^,^^19^ which was later shown to match chemical unfolding stabilities well.^20^ We have recently used a folding sensor based on a bacterial heat-shock response^21^ to select for variants with improved thermodynamic stability of an already stable protein.^22^ In line with this, we have shown that single substitution effects may be obtained by analyzing the results of a DMS experiment with many diverse multiple-substituted protein variants and suggested a global multi-mutant analysis (GMMA) for this task.^23^ GMMA rests on the observation that enhancing amino acid substitutions, although they have no individual phenotype in a given assay, can be identified by their ability to compensate deleterious substitutions, which have a phenotype. Combining the information of phenotype (e.g., growth/no growth) and genotype (mutations in the gene) in many multiply mutated variants allows for assignment of effects of individual substitutions, even if they do not display a phenotype on their own. Specifically, the current implementation is aimed at identifying generally enhancing substitutions characterized by additivity when combined.

We have previously developed an *in vivo* tripartite folding sensor based on the enzyme orotate phosphoribosyl transferase (OPRTase), encoded by the *pyrE* gene in *Eschericia coli* OPRTase, which is essential for pyrimidine biosynthesis.^9^ Cells defective in this enzyme can only survive on minimal medium if a pyrimidine source, e.g., uracil, is added. We engineered a circularly permutated variant of OPRTase as a folding sensor, termed CPOP, where POIs are inserted between the former N and C termini in the circular permutated enzyme. While the circular permutation is fairly unstable, it still complements a *pyrE* deletion; however, it becomes highly sensitive to the folding competence of the inserted POI. As a proof of concept, we enhanced the stability of a marginally stable designed protein (called dF106^24^) through conventional directed evolution. dF106 was created in an effort to computationally redesign the ubiquitous Rossmann fold of thioredoxins using Rosetta Design.^25^ This initial version of the protein was fragile but yielded a crystal structure close to the design target.^24^ Using the CPOP system, dF106 variants were selected, genetically resulting in a protein variant dF106-L11P-D83V, henceforth termed enhanced dF106 (edF106). This had a high stability with a folding energy of −48 kJ/mol. However, being extremely stable, this variant could not be improved further in the CPOP system because it had already reached the upper limit of the dynamic range in the CPOP selection.

In the present work, we have nevertheless further increased the stability of edF106 using CPOP in a DMS experiment on a library of more than 14,000 edF106 variants carrying, on average, 9 amino acid substitutions. GMMA estimates the additive effect of single amino acid substitutions on stability and function formulated as a fitness potential that relates to the assayed function.^23^ Because CPOP reports the folding competence of a variant, we will in this work refer to the estimated fitness potential as stability. Thus, by linking a sequence to the growth/no-growth phenotype, a stability effect was assigned to each of 374 substitutions. By introducing the nine top-ranking substitutions from this single experiment, the stability was enhanced to almost 70 kJ/mol with minimal structural changes. These results demonstrate how GMMA is capable of accurately identifying stabilizing substitutions for optimization beyond the dynamic range of the assay.

## Results and discussion

### Resilience toward mutations reflects thermodynamic stability

**Figure 1:**
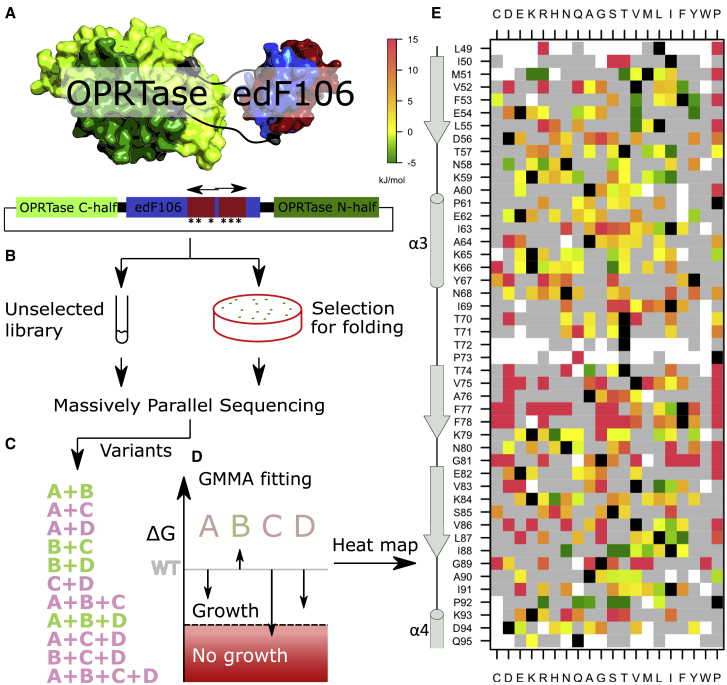
GMMA was used to analyze binary multi-mutant screening data from a genetic folding sensor and infer effect of individual amino acid substitutions (A) The enzymatic activity of the OPRTase (encoded by the pyrE gene) is essential for growth of *E. coli* on minimal medium, and, in the CPOP system, this is dependent of the folding of edF106.^9^ The C-terminal half (red) of the edF106 gene was mutated by PCR extension using long “doped” primers (horizontal arrows). (B) To screen variants for folding, the resulting plasmid libraries from unselected and selected cultures were subjected to MPS. (C) The combined effect of multiple substitutions (here labeled A–D), complementing (green) and non-complementing (pink), was determined by comparing reads from the two libraries. (D) The binary “growth/no growth” in which variants are classified is modeled on a continuous and additive stability scale. Energies consistent with the growth data of the variants are indicated by vertical arrows. (E) Heatmap of the stability effects consistent with the combination of the substitutions in the library. Black squares represent “wild-type” residues, and gray squares represent the 464 substitutions observed in the libraries but in insufficient representation to obtain robust stability estimates (see STAR Methods). White squares indicate that a variant is not present in the library.

We previously optimized the stability of the designed protein edF106 to the limit of the selective screen using the CPOP folding sensor.^9^ To map the effect of multiple mutations and push stability of edF106 beyond −48 kJ/mol, we generated variant libraries using semi-randomized oligonucleotides in the CPOP system (Figure 1A). If misfolded, this imposes uracil requirement on the cells and allows for genetic selection^9^ (Figure 1B). To generate a suitable dataset for application of the GMMA method, previous analysis had identified two key requirements for the variant library:^23^ (1) the library should encode a large number multiply substituted protein variants, each carrying different combinations of amino acid substitutions, all of which must be found in different contexts so that all are connected. In practice, this is achieved by having many-fold more variants than unique substitutions. (2) Similar to chemical unfolding experiments, the inactivation transition should be well probed. This is achieved by, on average, having a number of substitutions per variant that is close to the number of substitutions required to inactivate the protein (Figure S1).

Because the starting protein was already very stable, we opted for a high mutation frequency obtained by using randomly mutated (“doped”) oligonucleotides as degenerate primers^26^^,^^27^ with ∼10% error at each position of 77 and 75 base lengths (Figure 1A). These were designed to cover the C-terminal half of edF106, amino acid residues 48–97, and to act as long primers for PCR amplification and were assembled using USER cloning.^28^ This approach was also chosen because previous work suggested that error-prone PCR may result in an overrepresentation of mutations that occur in early PCR rounds, which is not ideal for GMMA.^23^ Despite the huge diversity possible, we applied measures to obtain a library of limited size (about 10,000–20,000 variants) such that essentially all variants could be covered by MPS with a reasonable depth (see STAR Methods). This was important because those variants present in the non-selected, but not in the selected, library were inferred to be non-functional in GMMA and should therefore be identified with high accuracy (Figure 1B). MPS of the library revealed 15,018 unique DNA variants with, on average, 13.4 mutations per gene (∼9 amino acid substitutions) after processing the data and applying a quality cutoff on the reads that eliminated sequencing errors (Figure S1A; Tables S1 and S2).

To identify the effect on stability of combinations of mutations using the CPOP system, the input library was plated on minimal medium to screen for growth (i.e., ura^+^ cells) and the resulting sublibrary sequenced by MPS. From the input library, 19% of the sequences were recovered after selection at 30°C (Figure S1E). We emphasize that the edF106 protein is exceedingly stable and that the stringency of the screen was calibrated accordingly. This was also reflected in the survival of the variants, which depended on the number of substitutions in a slightly sigmoidal fashion instead of an exponential decay (Figure S1A). This is consistent with previous observations and indicated that the genetic data could be modeled by the thermodynamic stability effects of the protein.^6^

### GMMA analysis discovers stability effects

The principle behind the GMMA analysis is that the effect of a given amino acid substitution is determined in very many different variant contexts and thus that the inferred effect is mostly independent of a specific context. Such generally stabilizing substitutions may be recognized by their ability to compensate other destabilizing substitutions, as earlier shown to be the case when considering double mutants.^17^ To illustrate this idea, consider two mildly destabilizing substitutions, A and D, a stabilizing substitution, B, and a deleterious destabilizing substitution, C (Figures 1C and 1D). Singly substituted variants of A and D both show wild-type-like growth (within noise), whereas they may both be inferred to be destabilizing from the observation that the double-substituted variant A + D does not complement growth. On the other hand, a variant, A + B + D, with an additional substitution, B, is observed to complement growth, from which we may infer that B is stabilizing because it rescues the inactivation by A and D. Using this concept, the effect of each individual substitution is determined from combinations with many others in a global fit to all variants.

GMMA was essentially computed as described previously.^23^ We used binary classification of folding and misfolding and relied on the robustness of GMMA to infer a quantitative stability effect. We validated this approach by comparing our previous GMMA analysis with one in which we had artificially made the data binary and find that the two are strongly correlated (Pearson correlation of 0.97; Figure S1F). By using a binary phenotype, GMMA simplifies to a logistic regression model with the particular fitting scheme described previously. Briefly, initial estimates were obtained using the mean-field approach followed by a global optimization using Levenberg-Marquardt damped least squares. Reliable stability effects could be assigned to 374 out of the 838 unique substitutions in the library based on the criteria that the substitution should be present in more than 40 sequences and have a standard uncertainty of less than 6.3 kJ/mol (Figure 1E).

**Figure 2:**
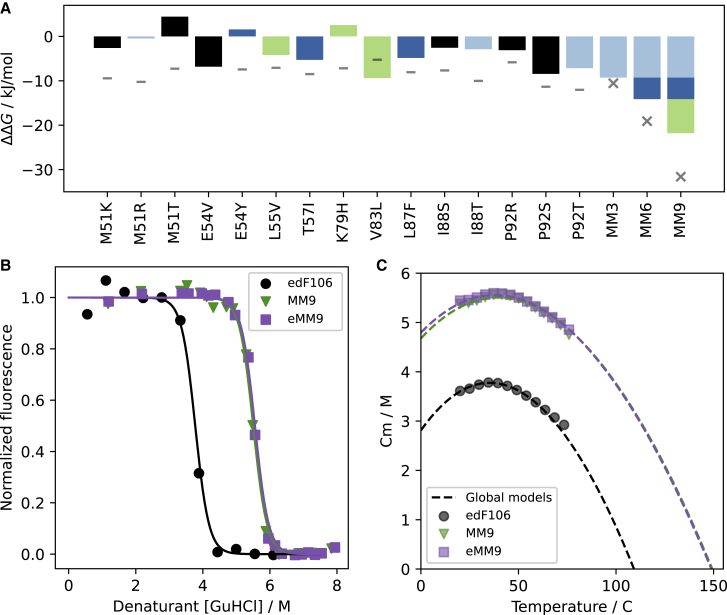
GMMA analysis successfully identifies substitutions that increase stability (A) Estimates of the change in stability for all single substitutions and combined multi-mutants (n = 1). The bars in the plot are colored in light blue, darker blue, and green to indicate their relationship with the MM3, MM6, and MM9 multi-mutants, respectively. Substitutions shown with black bars were not used in these multi-mutants because the GMMA analysis predicted that a more stabilizing substitution was available at the same position. The magnitude of the contribution of each substitution in the stacked bars plotted for MM6 and MM9 is only to guide the eye and contains no information of the individual contributions from each substitution. Horizontal gray lines indicate GMMA predictions, and gray “x” symbols indicate the expected stability of multi-mutants if substitutions were completely additive. (B) The average normalized fluorescence value of folding is shown as a function of denaturant for the starting point edF106 (black) and the stabilized multi-mutants MM9 (green) and eMM9 (blue). (C) The temperature dependence of the midpoint in the chemical denaturation, Cm, of the three proteins obtained by regular chemical denaturation fitting using isothermal slices of the NanoDSF data is plotted with the dashed line representing the global fit. The extrapolation of these fits to the absence of denaturant yields a melting point (temperature at which Cm = 0) increase to ∼150°C. Illustrations in (B) and (C) are from the MM9_3 and eMM9_3 replicates, and fits for the other replicates can be found in Figure S2.

The reference stability was estimated to −27.9 kJ/mol. This is substantially less stable than the value of −48 kJ/mol obtained from chemical unfolding,^9^ which indicates that the absolute scales of stabilities are not directly comparable. The global model may be inaccurate in this respect, but it is also likely that the thioredoxin domain is less stable in the fusion. Furthermore, with a binary phenotype, we may not expect the absolute stability scale to be accurate, and the present study only relies on the ranking of the substitutions (Figure S1F), although the absolute scale does seem relevant (Figure 2A).

### Stability measurements validate GMMA

To gauge the accuracy of our GMMA of the data from the folding sensor, we examined how well it could pinpoint the presumably rare stabilizing substitutions in edF106. As substitutions in proteins are on average mostly destabilizing,^29^^,^^30^^,^^31^ this was a stringent test of our analysis. We therefore introduced the 15 substitutions predicted to be most stabilizing by the GMMA into the isolated edF106 protein to measure their individual as well as combined effects on thermodynamic stability (Figure 2A). We used a “two-dimensional” unfolding approach, where denaturant unfolding is combined with a temperature scan,^32^ to measure the stability. For very stable proteins, this analysis arguably gives a more accurate measure of stability because the unfolding transition is probed at different temperatures, increasing the confidence in the extrapolation to a solution with no denaturant (Figures S2 and S3). The top 15 substitutions are found at nine distinct positions, with some positions having two or three substitutions that GMMA suggests are stabilizing. Thus, only nine could be combined to form the multi-mutant 9 (MM9) variant. To evaluate additivity, we constructed two additional multi-mutants with three and six of the nine top substitutions (MM3 and MM6). Interestingly, the multi-mutants show progressively increasing stability, whereas the individual stability effects vary more (Figure 2A). We compared the measured stability with the stability expected if all individual variant effects were additive and found that the total stability gains were 88%, 74%, and 69% in MM3, MM6, and MM9, respectively, of the expected. All the same, the MM9 variant had a stability of −68.6 ± 1.1 kJ/mol (ΔΔG = −21.8 ± 1.1 kJ/mol, n = 5) and an extrapolated melting temperature of 152°C ± 10°C (n = 5) (Figures 2B and 2C). While these values are somewhat uncertain due to the very long extrapolations to denaturant-free conditions, it is remarkable that this increase in stability was achieved in a single experiment of screening with a protein that was already very stable and that had hit the ceiling of the selection system used.

While 12 of the top 15 variants identified by GMMA were stabilizing in thermodynamic measurements of the isolated edF106 protein, three substitutions did not increase stability. Here, we must take into consideration that mutations were scored in the CPOP system, where insertion of target protein into a fusion with the sensor protein is not completely equivalent to that of the target, in this case a thioredoxin domain, on its own. Notably, the poorly predicted positions, E54Y, K79H, and M51T, are close to the N or C termini in the crystal structure of dF106 and are therefore in close physical proximity to the CPOP fusion site. Thus, mutations that could be stabilizing in the fusion might behave differently outside of the CPOP context. As two substitutions (E54Y and K79H) appeared to destabilize edF106 (Figures 2A and S2; Table S3) and the optimal substitution appeared not to have been chosen at all positions, we prepared an enhanced version of MM9, termed eMM9, in which the best possible substitutions at each position, according to the measurements of single variants, were chosen: M51K, E54V, L55V, T57I, V83L, L87F, I88T, and P92S. Within experimental error, however, we were not able to differentiate the stability of eMM9 from MM9 (Figures 2B and 2C) (ΔG = −69.5 ± 1.1, n = 4, p = 0.30 [two-sided independent t test]), again possibly due to the very long extrapolations to denaturant-free conditions. All things considered, effects on stability in the CPOP fusion are surprisingly well reflected in the isolated edF106 protein, and we note that including slightly destabilizing substitutions, as measured in single substitutions in edF106, did not abrogate the stabilization in the multi-mutant context.

### Crystal structures show increased similarity to the 1FB0 design template

**Figure 3:**
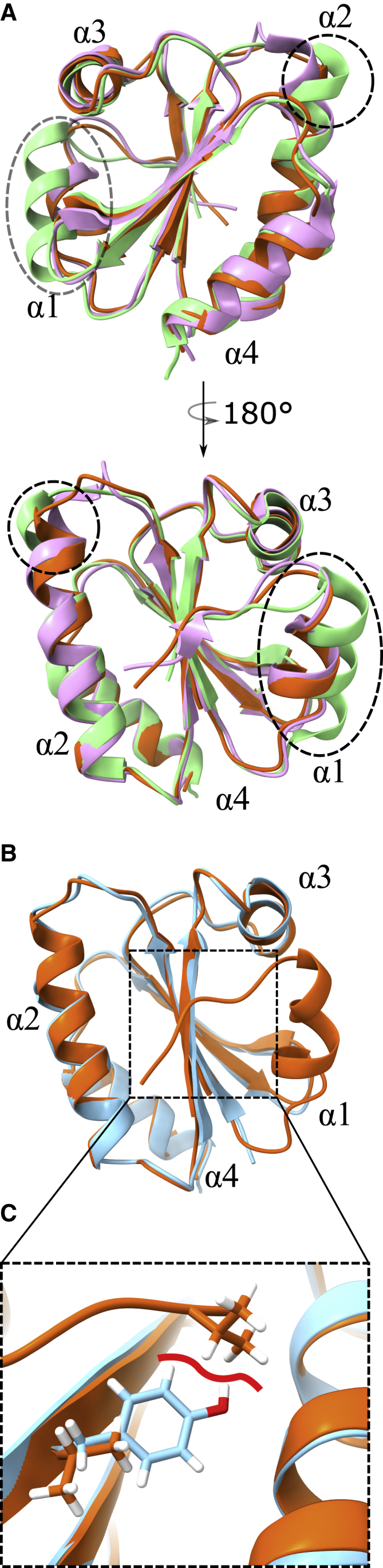
Crystal structure of the multi-mutants shows increased template similarity (A) Cartoon rendering showing dF106 (PDB: 5J7D) in green, eMM9 (PDB: 7Q3K) in orange, and the original spinach Trx design template (PDB: 1FB0) in purple. Bottom panel shows the structure flipped 180° around the vertical axis. Regions of structural rearrangement are highlighted within black circles. The mutated residues can be found drawn on the models in Figure S2. (B and C) Alignment of the structures of MM9 (PDB: 7Q3J, blue) and eMM9 (PDB: 7Q3K, orange) (B), with (C) showing in a magnified view that the placement of Val2 is incompatible with tyrosine at position 54.

To gain more insight into the effects of the mutations, we crystalized and determined the crystal structure of MM9 using diffraction data that extended to a resolution at 1.9 Å (Figure 3). Super imposition with the originally redesigned protein dF106 (PDB: 5J7D) showed a good fit with a root-mean-square deviation (RMSD) of 1.20 Å using the C_α_ positions. However, there was no visible electron density to fit the N-proximal residues (1–17). Instead, a strong crystal contact was present on the exposed surface and had likely moved the 17-residue helix (α1) out of the way (Figure S4). The crystals grew very slowly, typically within a month, with a low success rate out of 48 examined crystallization conditions. Only one or two would yield crystals indicative of a conformational change taking place in order to stabilize the crystal lattice. The similar construct of eMM9, however, readily formed crystals in several conditions, with the best dataset collected at 2.25 Å resolution. The complete model could readily be built into the electron density despite minor crystal twinning present in the dataset that challenged the data analysis (STAR Methods). This revealed an equivalent structure to MM9 with all C_α_ RMSDs at 0.70 Å, whereas when the structure was compared with dF106, the RMSD was measured to 1.83 Å. With the N-proximal helix visible (α1), the main difference between eMM9 and the original designed protein, dF106, is indeed found in this region where eMM9 showed much better agreement with its original design template spinach thioredoxin (PDB: 1FB0). Interestingly, the other main region of change is the start of helix 2 (α2)—the active site of the spinach thioredoxin—where MM9/eMM9 is again more akin to the natural protein. Thus, an all-C_α_ comparison of eMM9 showed an RMSD of 0.97 Å to spinach thioredoxin instead of 1.83 Å when compared with dF106 (Figure 3A). The structure also revealed that the destabilizing substitution E54Y presented in MM9, but not in eMM9, would probably be incompatible with the native conformation of Val2, which may have facilitated its increased dynamics following MM9 crystal formation (Figure 3C). That this clash happens close to the N-terminal further strengthens the idea that the discrepancy is likely an artifact from the fusion protein and not the GMMA method.

### Structure and sequence-based methods do not predict most stabilizing variants

**Figure 4:**
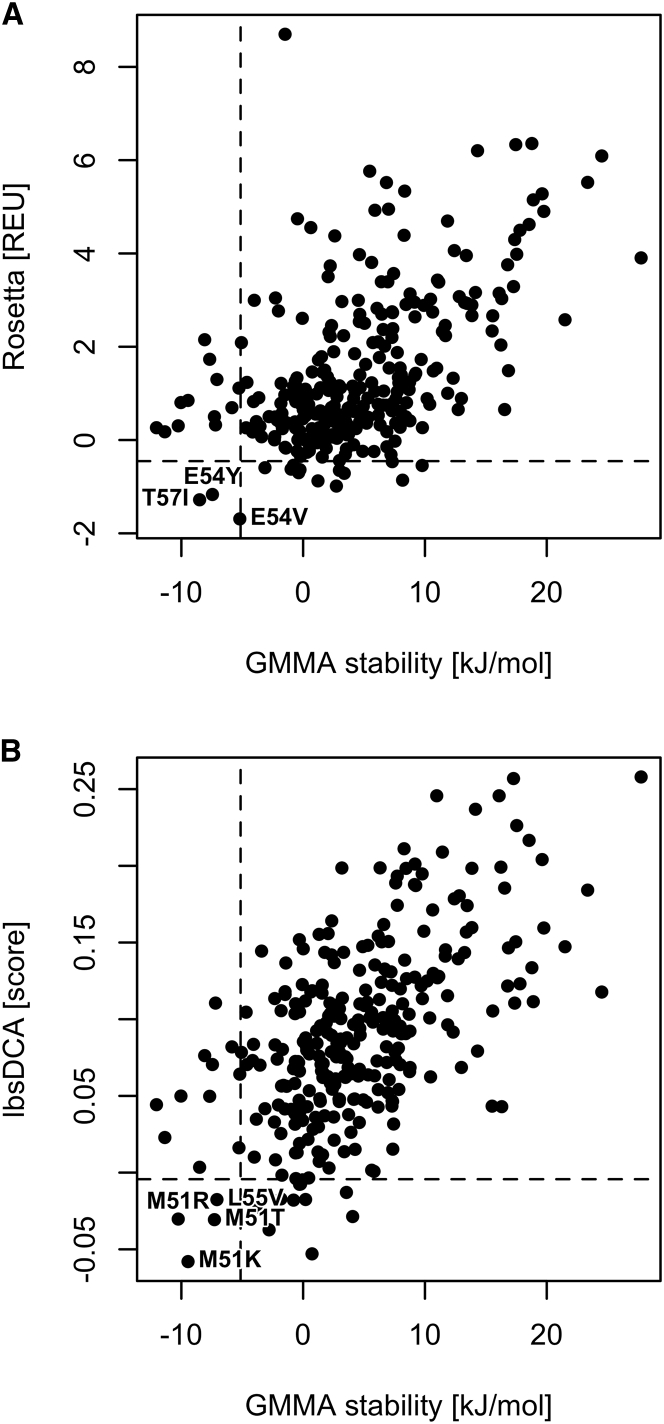
*In silico* models inadequately recapitulate the GMMA-inferred stabilities (A and B) The correlation between the GMMA and stability predictions using Rosetta molecular modeling (A), and an evolutionary conservation score, lbsDCA, in (B), are shown (see STAR Methods). Pearson correlations are 0.54 and 0.59 in (A) and (B), respectively. The dashed lines indicate the value of rank 15 for each measure and show the overlap in the top 15. In general, the *in silico* models do not reproduce the GMMA top 15 well, highlighting the benefit of genetic screening coupled with GMMA in stability design efforts.

In general, we found it difficult to rationalize why many of the substitutions stabilized the protein. We therefore asked whether it would have been possible to point them out as likely candidates beforehand. We calculated variant effects using two methods, structure-based stability calculations using Rosetta and a direct coupling analysis (lbsDCA) of a multiple sequence alignment (MSA) of natural thioredoxins, as such analyses have previously been shown to predict stability effects^22^^,^^33^^,^^34^ (Figure 4). While both methods gave rise to reasonable overall Pearson correlations of 0.54 and 0.59, respectively, they failed to identify many of the substitutions that were inferred to be stabilizing in our GMMA. Some of the best substitutions found here score poorly in Rosetta, e.g., P92T and P92S rank >50 and V83L, L87F, and L55V rank >150, and all five scored to be destabilizing. The MSA-based method, lbsDCA, in general performs better but still has V83L, P92S, and P92T with ranks >30 and E54V and L87F with ranks >100.

With 12 out of 15 substitutions confirmed to be stabilizing (Figure 2) and the zero-effect accurately reproduced (Figure S3), GMMA seems to identify stabilizing substitutions accurately. On the other hand, only 8 and 11 of the 15 top-ranking substitutions from Rosetta and lbsDCA are estimated to be stabilizing, respectively, and these effects are significantly smaller in magnitude as evaluated by GMMA. In particular, they identify only 3 and 4 of the GMMA top 15 ranking substitutions, respectively, and Rosetta rank 6 (K66I) and 13 (T74I) are estimated to be highly destabilizing and in the worst quartile of GMMA stabilities. This indicates that screening of multi-mutants coupled with GMMA is indeed a complementary and relevant method to obtain stability estimates for protein engineering.

### Computational evaluation of multi-mutant datasets as a general approach in protein engineering

In screening for protein/enzyme optimization the choice is often between (1) individually analyzing the full complement of amino acid substitutions throughout the open reading frame or (2) generation and screening of libraries of multiple random mutations using, e.g., error-prone PCR (epPCR). Option 1 has the advantage of being exhaustive because amino acid substitutions that require changing more than one base in a codon are more easily gauged. If there are indeed single substitutions, which offer clear enhancements, they are readily identified and implemented. Option 2, on the other hand, is less costly in library generation but will only access a smaller fraction of the possible amino acid substitutions. Also, because more than one substitution is typically introduced in each member of the library, significant time must be invested in identification of critical substitutions. In both cases, there needs to be screening headroom to actually measure enhanced stability/activity of variants beyond the current state of the protein. With GMMA, both this and the issue of variant identification are dealt with. Furthermore, substitutions identified by GMMA have a build-in additivity, and they may be particularly appropriate in the context of several other substitutions. Another virtue of GMMA is the fact that libraries do not need to (and, indeed, should not) be very large in order to cover all relevant substitutions because of the relatively high mutation rate applied. While we have here generated mutant libraries using doped oligo nucleotides, one could also have used epPCR instead. This choice does, however, depend on the screening stringency applied, which, as in the case of edF106, may require a higher mutation rate than is easily attainable by epPCR, to adequately sample the pass/no pass for the screen. Suitable deep sequencing can be done with entry-level MPS as library sizes need not be very large. Finally, while we have emphasized stability-enhancing substitutions here, the output of GMMA may also be useful for identifying subtle destabilizing substitutions, e.g., for targeted heat inactivation. As GMMA for binary readout screens simplifies to logistic regression, computational packages for such procedures can be adapted. For even easier access, enrichment or depletion of a given substitution in the multi-mutants after screening correlates with stability enhancement and decrease, respectively. Together, we anticipate that GMMA may be the method of choice for research laboratories or innovative small-to-medium-sized enterprises where access to resources may preclude exhaustive, but more costly, approaches.

### Conclusions

The GMMA approach shows remarkable efficiency in identifying the rare stabilizing substitutions of the model protein edF106, allowing us to improve the stability by close to 50% in a single iteration. The starting protein, edF106, can sustain growth even at the highest screening temperature 42°C in the CPOP selection system used, making it impossible to select for stability-enhancing substitutions by conventional directed evolution.^9^ Nevertheless, the selection for folding was here done under “mild” conditions (30°C), where the lower temperature imposes a less stringent conditions for protein folding. Thus, the GMMA approach allows us to identify stability-enhancing substitutions outside the dynamic range of the selection. While other measures can be taken to address this issue,^14^ we believe that the GMMA approach may be more robust. As GMMA only finds substitutions that are stabilizing in many variant backgrounds, stabilizing substitutions can be combined, suggesting that GMMA can efficiently guide optimization in the vast combinatorial space of the protein sequence. For the same reason, we expect that interrogating parts of target proteins and combining information from fragments provides useful and experimentally less demanding access to protein engineering. While the CPOP system has proven very successful here as a proxy for stability of a protein with no intrinsic function, GMMA can be applied to any system in which functional selection can be carried out in high throughput. Here, we have stabilized edF106 as an example, but we emphasize that the workflow should generalize to any other protein with a suitable screen for function.

### Limitations of the study

This study focused on the C-terminal half of edF106 in the effort of stabilizing the protein thermodynamically. Due to the length restrictions of the mutagenic oligonucleotides used for library creation, we were not able to cover the full protein, and one would need to develop individual sets of primers to cover the full sequence with this procedure. Nevertheless, as substitutions are likely additive throughout the full length, we would expect also to find stabilizing substitutions in the N-terminal half. The GMMA approach has, however, also been shown to work on libraries generated by epPCR mutagenesis,^23^ which does not have the same length limitations.

We have here not investigated the interplay of optimization of stability and function, e.g., catalytic activity of an enzyme. However, the method might in principle be able to improve both of these protein traits in parallel, which will also have valuable implications.

## STAR★Methods

### Key resources table

**Table:** 

REAGENT or RESOURCE	SOURCE	IDENTIFIER
**Bacterial and virus strains**		

*Eschericia coli* strain MRH205	Hansen et al.^35^	N/A
*Eschericia coli* strain MC1061	Casadaban and Cohen^36^	N/A
*Eschericia coli* strain BL21 (DE3)	NEB	#C2527H

**Chemicals, peptides, and recombinant proteins**		

PfuX7 polymerase	In-house purified	Nørholm^37^
USER enzyme mix	NEB	M5505S
T4 DNA ligase	Thermo Scientific	EL0011
Dpn1	Thermo Scientific	ER1701
BugBuster	Novagen	70584-3

**Critical commercial assays**		

GFX PCR DNA and Gel Band Purification Kit	Cytiva	28903470
E.Z.N.A.® Plasmid DNA Mini Kit	Omega	D6942-00
Hampton screen I and II	Hampton research	HR2-110 & HR2-112

**Deposited data**		

Crystal structure of MM9	This paper	PDB: 7Q3J
Crystal structure of eMM9	This paper	PDB: 7Q3K

**Oligonucleotides**		

“Doped” oligonucleotides	LGC Biosearch technologies	Table S5
1st PCR Illumina amplicon primers	Eurofins	Table S5

**Recombinant DNA**		

Plasmid pMMA010	Bjerre et al.^9^	N/A

**Software and algorithms**		

Analysis scripts and data	This paper	https://github.com/KULL-Centre/_2022_Norrild_GMMA_TRX and https://doi.org/10.5281/zenodo.7213166
GMMA	Johansson et al.^23^	https://github.com/KULL-Centre/_2022_Johansson_GMMA_GFP
R Project for Statistical Computing	N/A	https://www.R-project.org
Python 3.7	N/A	https://www.python.org/
biopython 1.73	Cock et al.^38^	https://biopython.org/
matplotlib 3.0.3	Hunter^39^	https://matplotlib.org/
matplotlib-venn 0.11.5	N/A	N/A
numpy 1.16.2	Oliphant^40^	https://numpy.org/
Pandas 0.24.1	McKinney^41^	https://pandas.pydata.org/
ProteinUnfolding2D	Hamborg et al.^32^	https://github.com/KULL-Centre/ProteinUnfolding2D
Rosetta	Park et al.^34^	https://www.rosettacommons.org
lbsDCA	Ekeberg et al.^42^	N/A
ChimeraX	Goddard et al.^43^	https://www.cgl.ucsf.edu/chimerax/
Inkscape	N/A	https://inkscape.org/

### Resource availability

#### Lead contact

Further information and requests for resources and reagents should be directed to and will be fulfilled by the lead contact, Dr. Jakob R. Winther (jrwinther@bio.ku.dk).

#### Materials availability

All unique/stable reagents generated in this study are available from the lead contact with a completed materials transfer agreement.

### Experimental model and subject details

#### Microbe strains

This study includes work done with *Escherichia coli* strain BL21 (DE3) for protein expression. *E. coli* strain MRH205 (MC1000 recA1 ΔpyrE::tetA/F’lacI^q1^ Z::Tn5 pro+) was used for assaying the function of CPOP constructs, and MC1061 (araD139 Δ(araA-leu)7697 Δ(lac)X74 galK16 galE15(GalS) lambda^−^ e14^−^ mcrA0 relA1 rpsL150(strR) spoT1 mcrB1 hsdR2).^9^

### Method details

#### Library construction

Libraries were constructed by using long “doped” oligonucleotides as primers for inverse PCR on a plasmid^9^ containing the fusion between the gene encoding CPOP sensor and a gene encoding edF106. Primers, obtained from LGC Biosearch technologies, contained a deoxyuracil at position 6 from the 5′ end so as to allow for annealing using a USER cloning approach.^44^ Primers were named based on the amino acid positions mutated; oligo [48:72] and oligo [74:97], and all positions not involved in the USER-cloning site, 71 and 69 bases respectively, were “doped” with 10% of the three non-wild-type nucleobases for random mutagenesis. A version of the plasmid pMMA010^9^ with edF106 inserted in the CPOP system was amplified in a PCR reaction using PfuX7 polymerase, which is compatible with the USER-cloning.^37^ 50 μL of PCR reaction mix [1x HF buffer (Thermo Fischer), 0.2 μM of each primer, 50 μM dNTPs, 0.15 ng/μL template plasmid] were prepared with 1 μL of in-house purified PfuX7. The reaction was run with initial denaturation at 98°C for 30 seconds and 30 cycles of 10 seconds at 98°C, 30 seconds at 62°C and 5 minutes at 72°C. A final 10 minutes at 72°C was employed to complete any unfinished product. Twenty 50 μL PCR reactions were pooled and the intended product was purified by gel band excision from a 1% (w/v) agarose gel. DNA was extracted from the gel with a gel band purification kit (GE healthcare) and eluted from the spin columns of the kit with 50 μL MilliQ water. 100 μL reactions were prepared for USER excision and ligation consisting of 85 μL purified PCR product, 10 μL 10X T4 DNA Ligase buffer (Thermo) and 5 μL USER enzyme mix (New England biolabs).^45^ The temperatures used for the reaction were 1 hour at 37°C for catalysis, 30 minutes at 25°C for dissociation of excised fragment and annealing consisting of 20 minutes at each of the following temperatures: 12°C, 11°C, 10°C, 9°C and 8°C. The solution was kept on ice before immediately adding 5 μL of T4 DNA ligase (Thermo) and 10 μL 5 mM ATP (VWR Life science). The solution was then incubated for 30 minutes at room temperature before heat inactivation of the ligase at 70°C for 10 min as recommended by the supplier. 2.5 μL Dpn1 (Thermo) were added before incubating the solution at 37°C overnight. Next day, the DNA was purified using the Illustra GFX PCR DNA and Gel Band Purification Kit (GE healthcare) and eluted in 20 μL sterilised MilliQ water.

#### Initial library transformation

10 μL of purified cloned DNA was used to transform electrocompetent MC1061 cells^36^ and the transformants were plated on four large 140 mm petri dishes to maximize colony separation, yielding an estimated 99,000 colonies. Colonies were scraped off the plates and collected by adding 5 mL sterile PBS buffer to each plate (20 mM phosphate and 150 mM NaCl, pH = 7.4). The density of the recovered cells where normalised to OD_600_ = 3 before isolating plasmids using a mini prep kit (Omega) for each plate and eluting in 50 μL TE buffer (10 mM Tris-HCL and 1 mM EDTA, pH = 7.3). To normalise the number of variants obtained from each purification, the purified plasmids were mixed in equimolar volume based on their absorbance at 260 nm.

#### Retransformation

The plasmid libraries were transformed into the selection strain by using 5 ng of the purified and mixed plasmid library to transform electrocompetent MRH205 cells.^9^ Ten-fold dilution of the transformed cells were plated on a 140 mm LB agar plate with 100 μg/mL ampicillin and 50 μg/mL kanamycin for a limited library size of estimated 53,600 colonies. Cells were collected using the same protocol as for the initial library transformation. A freeze stock was prepared for each library with 800 μL cell suspension and 200 μL of 87% (v/v) glycerol. 50 μL aliquots were made from the freeze stock for screening of the libraries. Plasmids from single colonies of the library were purified and Sanger sequenced to confirm correct assembly of the library. Sequences appeared clear and uniform, suggesting that none of the transformants tested carried significant levels of more than one variant.

#### Screening of library

The cell library was diluted 100,000-fold in PBS buffer and plated on three 140 mm selective medium plates at 30°C ensuring no more than 80 CFU/cm^2^ but a five-fold sampling depth of the library. After 22.5 hours incubation, plasmids from the plates were purified similarly to the collection of the initially transformed libraries. The concentration of DNA in the mini preps were normalised by dilution in TE buffer before mixing equal volumes of the solutions. In parallel, the library was also grown over night in 5 mL LB with 100 μg/mL ampicillin and 50 μg/mL kanamycin before purifying the plasmids of the full library using the same mini prep kit.

#### Massively parallel sequencing

Sequencing of the purified plasmids were done with paired end amplicon sequencing protocol^46^ on one third of a Illumina MiSeq run using the version 3 kit with 600 cycles. The mutated part of the protein and 70 base pairs flanking region in each direction was sequenced. Amplicons with Illumina Nextera primers (361 base pairs) were produced by a PCR reaction with two HPLC purified primers with one part complementary to the plasmid and the Nextera sequence as a 5′ overhang. 25 ng template were used for the 25 μL PCR mix using HiFi Pfu polymerase (PCR biosystems). The reaction (Initial denaturation: 1 min at 95°C. Cycle: 15 s at 95°C, 15 s at 55°C and 1 min at 72°C) was run for a minimum amount of cycles (12 cycles) as to reduce PCR chimeras.^47^ The final elongation step was omitted to reduce the amount of chimeras.^48^ Amplicons from the first PCR were purified from 20 μL using AMPure XP magnetic beads (Beckman Coulter) and eluting in 40 μL elution buffer (Zymo kit). 2 μL of purified amplicons were used as templates for the second PCR to attach indexing primers to the Nextera adapters (total of 429 base pairs), including one reaction without template as a negative control. The PCR reaction was run for 15 cycles and was checked for uniform bands on a gel. 30 μL were purified using the magnetic beads (AMPure XP, Beckman Coulter) and eluted in 40 μL elution buffer (Zymo kit). The concentrations of the samples were then normalised with SequalPrep Normalization Plate (Invitrogen) and eluted in 20 μL elution buffer (Zymo kit). The amplicons were pooled and then cleaned and concentrated with the DNA Clean & Concentrator kit (Zymo). The concentration of the DNA in the eluate was quantified with Qubit and the sample was then diluted to 4.5 nM. The sample was denatured and diluted as described by the Illumina protocol for the MiSeq system. 200 μL sample were mixed with 400 μL of other samples for the run before removing 30 μL of the pooled samples and adding 30 μL PhiX (5% spike) as internal control. 1.35–1.5 million reads were collected totalling ∼3 million paired end reads before and after selection (Table S1).

#### Processing the paired end reads

Data from the sequencing were demultiplexed and trimmed to the start of the plasmid coding sequence. Filtering was done with a custom python script (Filtering.py) that checked for full complementation of the mutated area plus 10 base pairs in each direction. Also, the length of the amplicon was required to match the expected size. The area was then compared to the template sequence using a custom script (GenerateMutfile.py) to compress the sequences to a list of mutations. Identical sets of mutations were then counted (SeqCount.py), resulting in a file with the mutations of the sequences and how many times they were read. For each sequencing pool, a cut-off was chosen to eliminate noise from the dataset based on the mutation rate in the 2 × 10 base pair region immediately down and upstream of the mutated area.

#### GMMA

The genotype counts were aggregated per amino acid variant and 226 complementing variants that were not observed in the input library were discarded. No pseudo-counts were used. Variants with any counts above the cut-off values were considered complementing in the binary readout. This resulted in 838 unique amino acid substitutions combined in 14.887 protein variants holding on average of 9.0 substitutions and 18.5% variants that complement growth in CPOP. The following GMMA was conducted according to the previously published protocol.^23^

Using the mean-field approach,^23^ initial stability estimates could be obtained for all 838 unique amino acid substitutions based on the 10,235 variants that did not contain non-sense mutations (4622) or substitutions that like-wise appeared irreversible fatal (30). Of these, relatively few (194) substitutions were observed in only active or only inactive variants.

For the global analysis, a network analysis found that all substitutions were connected, i.e. that no subset of substitutions only occurred together and never with the rest of the substitutions. This test was important because only a connected network of substitutions can inform the global analysis. Only 54 of the 838 unique substitutions only occurred in a single variant, i.e. are hanging substitutions that do not inform the global analysis.

The GMMA error analysis was slightly different from previously described.^23^ We did not obtain errors on the estimated stability effects since uncertainties were not determined in the binary readout from the CPOP screen. For filtering of inaccurately estimated effects, we simply required that a substitution should be observed in at least 40 different variants and have a fitting standard uncertainty of 6.3 kJ/mol or better. This resulted in 293 accurately estimated effects. Requiring that a substitution has been observed in at least 40 variants is rather conservative compared to previous applications of GMMA and was here selected for robustness against the potentially noisy experimental data. Additionally, 81 substitution effects estimated to destabilize more than the reference stability but with higher uncertainty were included as destabilizing resulting in 31 stabilizing, 79 neutral, 264 destabilizing and 464 unknown substitution effects.

#### Protein purification

Genes encoding single substitution variants of edF106, derived from the GMMA, were custom synthesized by Twist Bioscience cloned into pET-29b(+) using restriction sites Ndel and XhoI flanking the His_6_ sequence. This resulted in a C-terminal insertion of leucine and glycine before the His_6_-tag. Plasmids were solubilized in TE buffer to 10 ng/μL and 2 μL were used to transform chemically competent BL21 (DE3) cells which were subsequently plated on LB medium with 50 ng/mL kanamycin. Starter cultures were prepared by using single colonies to inoculate 800 μL LB medium with 50 ng/mL kanamycin in a 48 well plate format and incubating over night at 37°C. 2 mL of TB-5052 auto induction medium were inoculated with 20 μL overnight culture and grown for 24 hours at 25°C in 24 deep well plates. TB-5052 is a phosphate buffered medium containing salts and metals for optimized protein expression and a mix of glucose, lactose, and glycerol for auto-induction of the Lac-promoter once the culture as reached appropriate density.^49^ Next day, cells were harvested in the plate by centrifugation at 4,250 g for 20 minutes. The supernatants were removed and 500 μL lysis buffer pH = 7.0 (50 mM phosphate, 300 mM NaCl, 20 mM imidazole, and 1x BugBuster (10x solution from Novagen)) were added to the cell pellets. The plate was incubated while shaking for 25 minutes for lysis before pelleting the insoluble part of the lysate by centrifugation at 4,250 g for 40 minutes. The supernatants were transferred to a 96 well filter plate with 250 μL 50% slurry of nickel-NTA beads (Thermo) in each well. After wash with a total of 2 mL buffer with 20 mM imidazole the proteins were eluted in 200 μL buffer with 400 mM imidazole. To remove the imidazole, the IMAC eluates were buffer exchanged on Nap5 columns (Cytiva) and the peak fractions were eluted in 200 μL analysis buffer (50 mM phosphate and 150 mM NaCl, pH 7.0). 1 μL protein solution was mixed with 19 μL 0.1% (v/v) TFA for mass spectrum analysis to confirm mutant identity.

#### Protein stability measurements

Two-dimensional denaturation and renaturation of the proteins was measured using the Prometheus NT.48 (NanoTemper) using a heating and cooling ramp of 1°C/min. For the preliminary estimation of stabilities, twelve 40 μL samples with equally spaced guanidine hydrochloride (GuHCl) concentration were prepared from two 250 μL solutions, one having 6 M GuHCl, with the same protein concentration (≥5 μM) using a pipetting robot (1000G Andrew Alliance) for consistency. After loading the samples into the capillaries, the ends were sealed with high vacuum grease (Dow Corning) to avoid evaporation during the experiment. Folding and refolding curves were acquired. 5 μL sample without GuHCl were analysed on SDS-PAGE to check that the proteins were pure. The data obtained were fitted using dTrx_stability.ipynb based on the ProteinUnfolding2D.py python module.^32^ After fitting individual m-values to each dataset in the initial fit, the average m-value was subsequently used for all dataset to get more comparable ΔΔG-estimates. Samples without GuHCl and samples judged to be outliers were not used for the fits for all datasets. For absolute estimation of the stability of MM9 and eMM9, samples were prepared with increased density in the transition region.

#### Crystallization of MM9 and eMM9

The MM9 construct was concentrated to 10 mg/mL for crystallization experiments using the Hampton screen I and II (Hampton Research). Crystal drops were mixed using 1 μL of protein and 1 μL precipitant solution in 24-well plate as hanging drops on siliconized glass cover-slides. The wells were sealed with vacuum grease (Dow Corning high-vacuum silicone). Plates were incubated at room temperature. Initial crystals of MM9 appeared after approximately a month and grew to a maximal size of 100 × 100 × 300 μm, crystal condition: 0.2 M NaOAc, 0.1 M Tris-HCl (pH 8.5) and 30% (w/v) polyethylene glycol 4000 (PEG4000). Crystals were harvested using mounted CryoLoops (Hampton Research) and flash frozen in liquid nitrogen. Cryo protection was performed by quick dipping the crystal 0.1 M NaOAc, 0.05 M Tris-HCl (pH 8.5), 15% (w/v) polyethylene glycol 4000 (PEG4000) and 20% (v/v) Glycerol. The data were collected from crystals cooled to 100 K on a PILATUS detector at BioMax (MAX-IV, Lund, Sweden). A full sweep of 360° data was collected with an oscillation degree of 0.1°, with 0.050s exposure, at 12,650 eV. Complete data set was processed from 200° (2000 images) with xia2^50^ using the dials pipeline option to account for the weak ice rings (see Table S4).

The eMM9 crystals were obtained in a similar procedure, but initial crystals of eMM9 appeared in seven days and grew to a maximal size of 100 × 100 × 300 μm. The best eMM9 crystals were grown using a reservoir solution of 0.2 M ammonium sulfate, 0.1 M sodium acetate, pH 4.6, 25% (w/v) Polyethylene glycol 4.000. Crystals were harvested using mounted CryoLoops (Hampton Research) and flash frozen in liquid nitrogen, cryo protection was performed by quick dipping the crystal in 0.2 M ammonium sulfate, 0.1 M sodium acetate, pH 4.6, 25% (w/v) Polyethylene glycol 4.000, 20% (v/v) Glycerol. The data were collected from crystals cooled to 100 K on a PILATUS detector at BioMax (MAX-IV, Lund, Sweden). A full sweep of 360° data was collected with an oscillation degree of 0.1°, with 0.050s exposure, at 12,650 eV. Complete data set was processed from 180° (1800 images) with xia2 using the dials pipeline option to account for the weak ice rings (see Table S4).

Molecular replacement using the program Phaser^51^ was used to solve the phases using the structure of dF106 (PDB: 5j7d) as an initial search model. The initial model was build using the AutoBuild wizard within the PHENIX package,^52^ and for eMM9 the twinning operator h,-h-k,-l was used to account for the crystal twinning. The structure was further manually refined using phenix.refine.^53^ Final model building was performed in Coot.^54^ Data collection and refinement statistics are summarised in Table S4.

#### Calculation of rosetta stabilities

These calculations were carried out as described previously.^55^ Briefly, we used the Cartesian ΔΔG protocol^34^ and the X-ray structure of dF106 (PDB: 5J7D). During the initial relaxation of the structure, a res-file was used to introduce L11P and D83V in order to obtain a model of edF106 to be used with the cartesian_ddg application.

#### Calculation of lbsDCA conservation scores

These calculations were carried out as described previously.^55^ Briefly, we used a statistical analysis of a multiple sequence alignment (MSAs) generated by HHBlits^56^ using the sequence edF106 as target. We used a modified version of the lbsDCA^42^ that includes both positional and pairwise conservation of amino acids. While originally designed to identify contacts between residues, we use the energy potential generated by the algorithm to evaluate the log-likelihood difference between the wild type and the variant sequences.

### Quantification and statistical analysis

A two-sided independent t-test was used to determine that no statistical significant (5% significance level) difference could be observed between the thermodynamic folding stability of proteins MM9 and eMM9. n = 5 and n = 4, respectively, and represent independent replicates of stability measurements (see data in Table S3).

## Data Availability

• Crystallography structures have been deposited in PDB with accession numbers 7Q3J and 7Q3K and are publicly available as of the date of publication. • All original code has been deposited at GitHub: https://github.com/KULL-Centre/_2022_Norrild_GMMA_TRX (https://doi.org/10.5281/zenodo.7213166) and is publicly available as of the date of publication. • Any additional information required to reanalyze the data reported in this paper is available from the lead contact upon request.

## Supplemental information

Document S1. Tables S1–S5 and Figures S1–S4

Document S2. Article plus supplemental information
