# Supplementary material for: Increasing protein stability by inferring substitution effects from high-throughput experiments
Source: Cell Rep Methods. 2022 Nov 14;2(11):100333. doi: 10.1016/j.crmeth.2022.100333 (PMC9701609; doi:10.1016/j.crmeth.2022.100333)
Supplement: Document S1. Tables S1–S5 and Figures S1–S4 [file mmc1.pdf]

Cell Reports Methods, Volume *2*

Supplemental information

Increasing protein stability

by inferring substitution effects

from high-throughput experiments

Rasmus Krogh Norrild, Kristoffer Enøe Johansson, Charlotte O'Shea, Jens Preben Morth, Kresten Lindorff-Larsen, and Jakob Rahr Winther

Supplementary information

Supplementary tables

Sample	Total reads	Reads passed (%)	Unique DNA sequences after cut-off	Unique protein sequences after cut-off
[48:97] input	1,357,174	356,577 (26.3)	15018	14978
[48:97] 30deg	1.494,800	654,314 (43.8)	3009	2980

Supplementary Table 1, related to Figure 1: Sequencing data from the libraries and for the filtering of sequences based on how many times they were observed. The lower fraction of reads passing the filters for the input libraries in part reflect species that were cloned incorrectly and therefore did not have the correct length.

Sample	[48:72] area	[74:97] area	Total	Not mutated
[48:97] input	6.61	6.74	13.40	0.05
[48:97] 30deg	5.34	4.90	10.27	0.03

Supplementary Table 2, related to Figure 1: The average mutations per sequence in each of the samples. The mutation G148T (at the DNA level) is omitted from this count because it was a technicality of the cloning procedure. Mutations in the regions originating from the mutated oligonucleotides are counted separately. The [74:97] oligonucleotide is slightly less mutated than then [48:72]. The last column shows that very few mutations were seen outside of the mutated area.

Protein	ΔG (25°C)	m-value	Tm	ΔHs	ΔCp	X^2	ΔΔG
edF106	-46.83	12.67	109.25	477.63	5.81	2.29E+08	0.00
M51K	-49.43	12.67	103.54	555.29	7.52	1.68E+08	-2.61
M51R	-47.32	12.67	105.39	507.02	6.55	2.40E+08	-0.49
M51T	-42.39	12.67	102.24	464.82	6.22	4.41E+08	4.44
E54V	-53.65	12.67	113.80	529.29	6.12	6.63E+08	-6.82
E54Y	-45.26	12.67	110.98	448.62	5.29	2.00E+07	1.57
L55V	-51.01	12.67	110.65	502.96	5.91	1.18E+08	-4.18
T57I	-52.12	12.67	119.04	485.83	5.23	1.52E+08	-5.29
K79H	-44.29	12.67	115.03	432.14	4.93	3.37E+07	2.54
V83L	-56.21	12.67	108.30	579.21	7.14	2.96E+08	-9.38
L87F	-51.68	12.67	110.77	504.76	5.88	5.91E+07	-4.85
I88S	-49.38	12.67	103.60	527.14	6.86	2.98E+08	-2.55
I88T	-49.72	12.67	109.64	488.73	5.76	2.03E+08	-2.90
P92R	-49.94	12.67	110.75	499.32	5.93	9.08E+08	-3.11
P92S	-55.28	12.67	110.16	549.43	6.52	4.06E+08	-8.45
P92T	-53.98	12.67	117.50	499.43	5.39	5.45E+08	-7.15
MM3	-56.12	12.67	113.39	532.98	5.99	9.19E+07	-9.29
MM6	-60.95	12.67	132.59	487.43	4.34	6.97E+07	-14.13
MM9	-67.58	12.67	143.97	499.94	3.98	5.33E+07	-20.75
MM9_0	-67.68	12.67	170.86	431.53	2.73	1.49E+09	-20.85
MM9_1	-69.76	12.67	151.34	505.07	3.84	1.30E+09	-22.93
MM9_2	-69.81	12.67	145.86	527.09	4.24	1.49E+09	-22.98
MM9_3	-68.38	12.67	149.36	511.37	4.02	6.32E+08	-21.55
eMM9_0	-67.97	12.67	157.09	470.13	3.36	2.21E+08	-21.14
eMM9_1	-70.34	12.67	145.17	530.95	4.28	1.53E+09	-23.51
eMM9_2	-70.24	12.67	146.79	519.73	4.09	7.43E+08	-23.42
eMM9_3	-69.27	12.67	148.92	510.02	3.97	4.27E+08	-22.44

Supplementary Table 3, related to Figure 2: Fitted parameters of combined temperature and denaturant unfolding of purified proteins for Figure 2a, b and c. The m-value was kept constant (see Methods). ΔG (25°C) and ΔΔG values were derived from the other fitted parameters. ΔG, ΔH and ΔΔG are in kJ/mol, the m-values are in kJ/(mol·M), T_m is in K, and ΔC_p in kJ/(mol·K).

	MM9 (PDB: 7Q3J)	eMM9 (PDB: 7Q3K)
Wavelength	0.98	0.98
Resolution range	25.93 - 1.9 (1.968 - 1.9)	35.42 - 2.25 (2.33 - 2.25)
Space group	C 1 2 1	P 31
Unit cell	58.89 45.66 72.88 90 92.16 90	70.85 70.85 75.32 90 90 120
Total reflections	53102 (5297)	148581 (14130)
Unique reflections	14904 (1490)	20043 (1984)
Multiplicity	3.6 (3.6)	7.4 (7.1)
Completeness (%)	96.53 (95.69)	99.85 (99.90)
Mean I/sigma(I)	22.28 (2.24)	14.13 (2.90)
Wilson B-factor	28.66	
R-merge	0.063 (0.32)	0.073 (0.53)
R-meas	0.074 (0.37)	0.079 (0.58)
R-pim	0.039 (0.19)	0.029 (0.21)
CC1/2	0.997 (0.755)	1 (0.97)
CC*	0.999 (0.928)	1 (0.99)
Reflections used in refinement	14893 (1487)	20043 (1984)
Reflections used for R-free	759 (74)	1050 (134)
R-work	0.19 (0.28)	0.29 (0.39)
R-free	0.24 (0.30)	0.30 (0.37)
CC(work)	0.96 (0.75)	0.9 (0.64)
CC(free)	0.91 (0.84)	0.87 (0.61)
Number of non-hydrogen atoms	1532	2583
macromolecules	1445	2541
ligands	28	10
solvent	75	32
Protein residues	176	312
RMS(bonds)	0.012	0.011
RMS(angles)	1.17	1.15
Ramachandran favored (%)	98.84	93.46
Ramachandran allowed (%)	1.16	6.54
Ramachandran outliers (%)	0.00	0.00
Rotamer outliers (%)	0.00	0.00
Clashscore	5.09	14.07
Average B-factor	39.22	62.00
macromolecules	39.06	61.96
ligands	50.08	58.67
solvent	40.64	65.54
Number of TLS groups	12	12

Supplementary Table 4: Crystal structure statistics, related to Figure 3. Statistics for the highest-resolution shell are shown in parentheses.

Name	Sequence
edF106 open reading frame	ATGGTACTGGATGTAACGAAAGATCACTGGCTGCCCTACG<br>TATTACTCGCTCAACTGCCGGTCATGGTGTTGTTCCGTAA<br>AGATAACGACGAAGAGGCCAAGAAGGTTGAGTATATTGTG<br>CGCGAACTGGCGCAGGAATTTGACGGTCTGATCATGGTTT<br>TCGAGCTGGACACGAACAAGGCACCGGAGATCGCGAAAAA<br>GTACAATATCACCACCACCCCGACTGTCGCATTTTTCAAA<br>AATGGCGAGGTCAAGAGCGTTCTGATTGGCGCGATTCCAA<br>AAGACCAGCTGCGTGATGAAATCCTGAAATATCTGGGTCA<br>CCATCATCACCATCAC
oligo [48:72] (doped)	accccuACTGTCGCATTTTTCAAAAATGGCGAGGTCAAGAGCGTTC<br>TGATTGGCGCGATTCCAAAAGACCAGCTG
oligo [74:97] (doped)	aggggu GGTGGTGATATTGTACTTTTTCGCGATCTCCGGTGCCTTG<br>TTCGTGTCCAGCTCGAAAACCATGATCAGAC
Illumina amplicon primer forward	TCGTCGGCAGCGTCAGATGTGTATAAGAGACAGccgtaaagataac<br>gacgaagaggc
Illumina amplicon primer reverse	GTCTCGTGGGCTCGGAGATGTGTATAAGAGACAGtcgatgaactga<br>cgttggtacgg

Supplementary Table 5, related to Figure 1 and STAR Methods: DNA sequences. For "doped" oligonucleotides, upper case letters symbolize mutagenized positions:

Supplementary figures

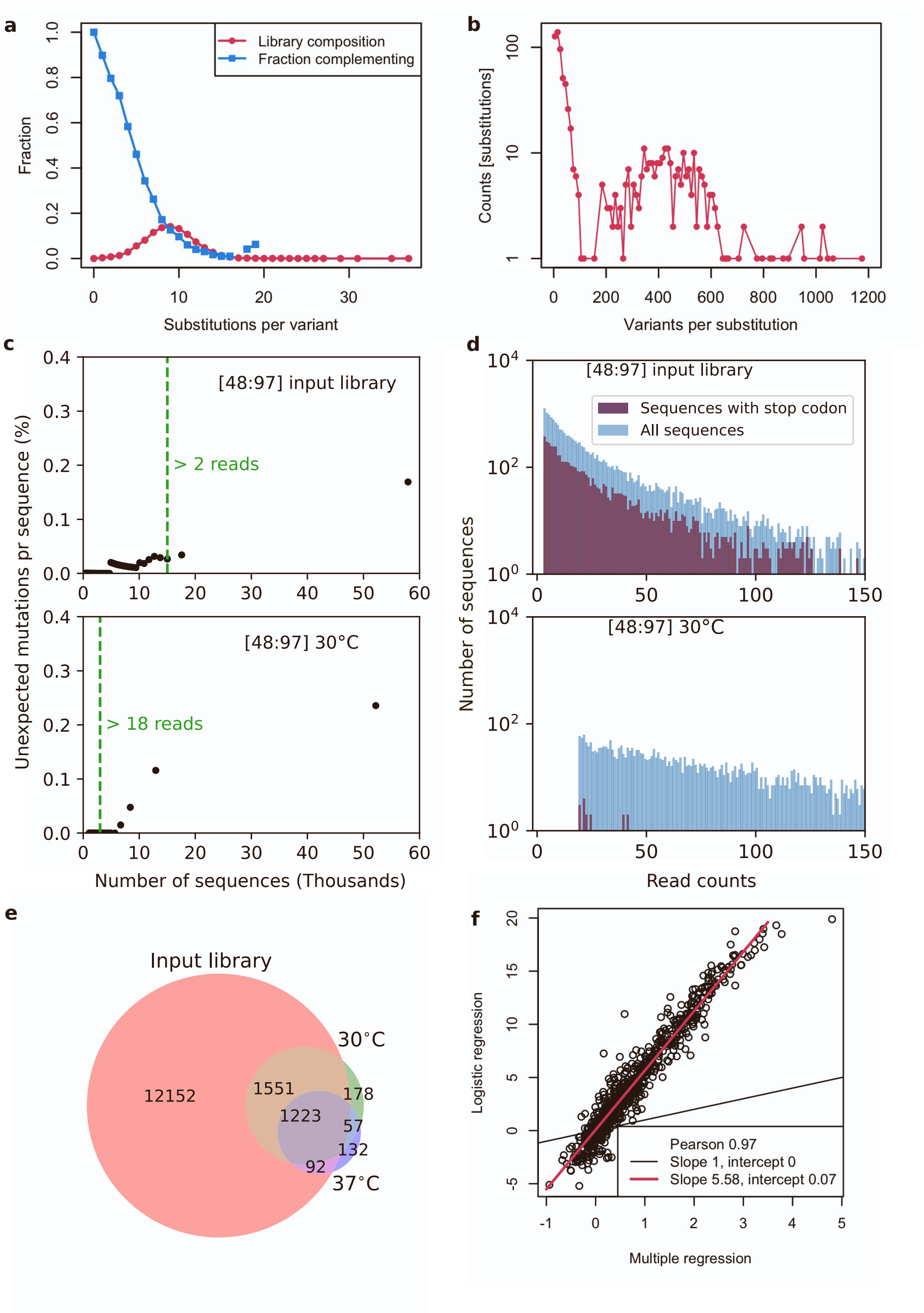

Figure S1. Mutant library characteristics. Related to Figure 1 and STAR Methods. a, The broad distribution of muti-mutants in the variant library (red) covers the complementation profile (blue) sufficiently to give a total of 19% complementing variants. Variants with 9 substitutions are most abundant and approximately half of the 5-mutants complement in the assay. **b**, The library stategy using long mutated primers is shown to result in a relatively narrow peak between 200 and 600 variants per substitution which is a relatively homogeneous amount of data per estimated parameter in GMMA. Effects of substitutions observed in less than 40 different variants are assumed to inaccurately estimated in the error analysis. **c**, Sequencing data cut-off is choosen to eliminate noise with plots showing the rationale for choosing cut-offs for the number of sequencing reads required to remove sequencing noise. Cut-off stringency increases from right to left, because fewer sequences are left when a more stringent cut-off is used. The metric used to determine the minimum number observations of a sequence required to eliminate sequencing noise and thus be included in the downstream analysis (cut-off level) is the average number of mutations in the 10 base pairs immediately up- and downstream of the region intentionally mutated. Here, these are termed “unexpected mutations”. The rate of such mutations were recorded for each cut-off value (>1,>2,>3…>N reads incresing right to left) plotted as single points from right to left (black dots) on the graphs, based on the remaining number of sequences (x-axis). Green text and vertical bars indicate the cut-offs chosen for the analysis. **d,** Sequences with stop codons were highly depleted after selection. Histograms of readcounts for each sequence identified by MPS from the library [48:97] in blue, with the fraction of sequences containing a stop codon in red. **e,** Venn diagram showing the amount of reads found in the different libraries before, and after selection at 30°C and 37°C. **f,** Using binary data for GMMA is appropriate as shown on GMMA of GFP variants from a previous study (Johansson et al., 2021). The plot shows the correlation between estimated stability effects for the original continuous fluorescence readout (multiple regression) and a binary readout generated from the same data (logistic regression). The correlation shows that for GFP, the ranking and zero-point (intercept) is preserved. The results also show that the scale of GMMA changes when the assay is made binary.

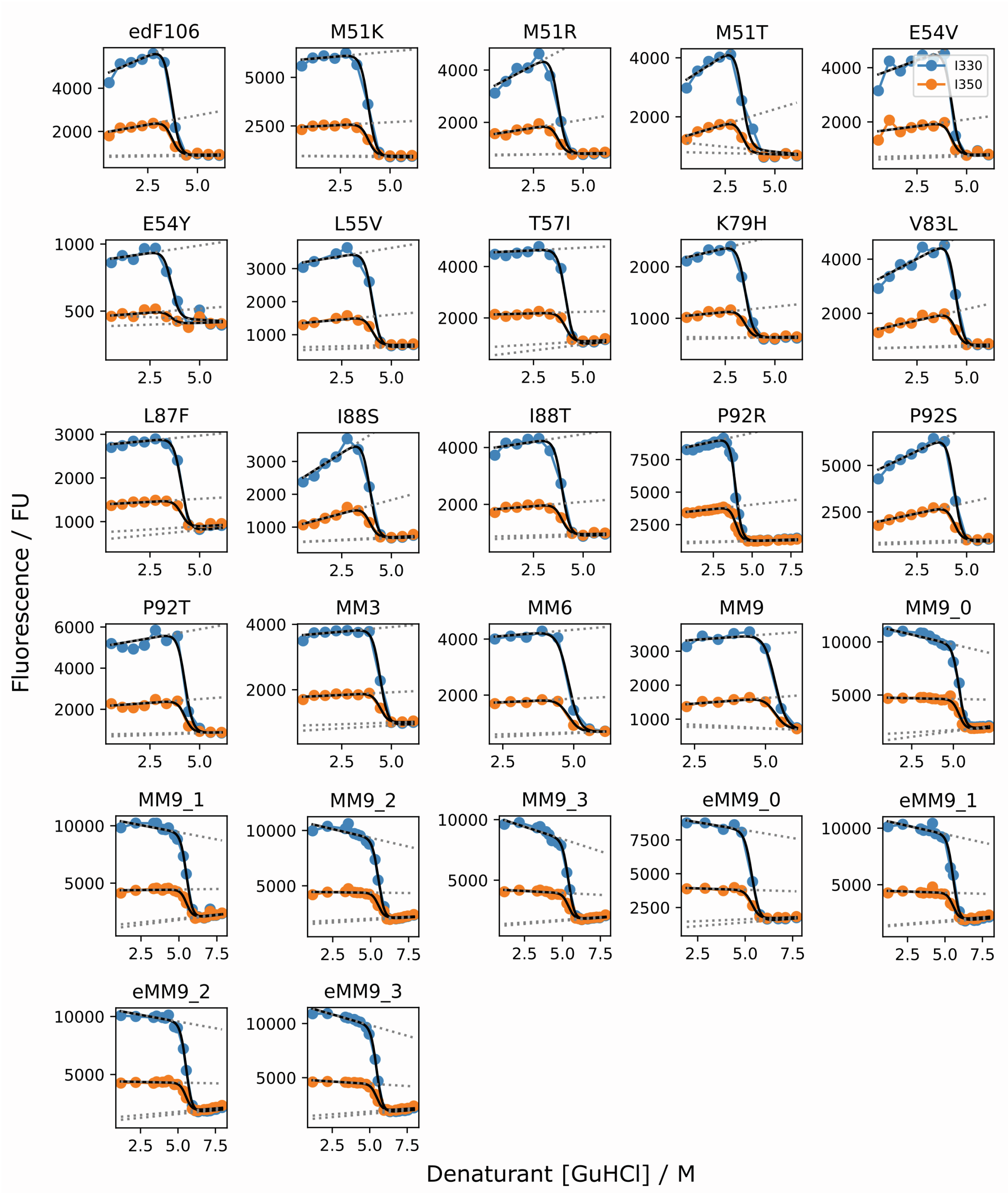

Figure S2: Isotherms from two-dimensional fitting of variants, Related to Figure 2. Raw data for fitting global denaturation using denaturant and temperature shown only in the denaturant dimension. The data from the fluorescence intensity at 330 and 350 nm obtained on the Prometheus NT.48 for folding and refolding was fitted globally. The baselines of the fits are shown in grey dots.

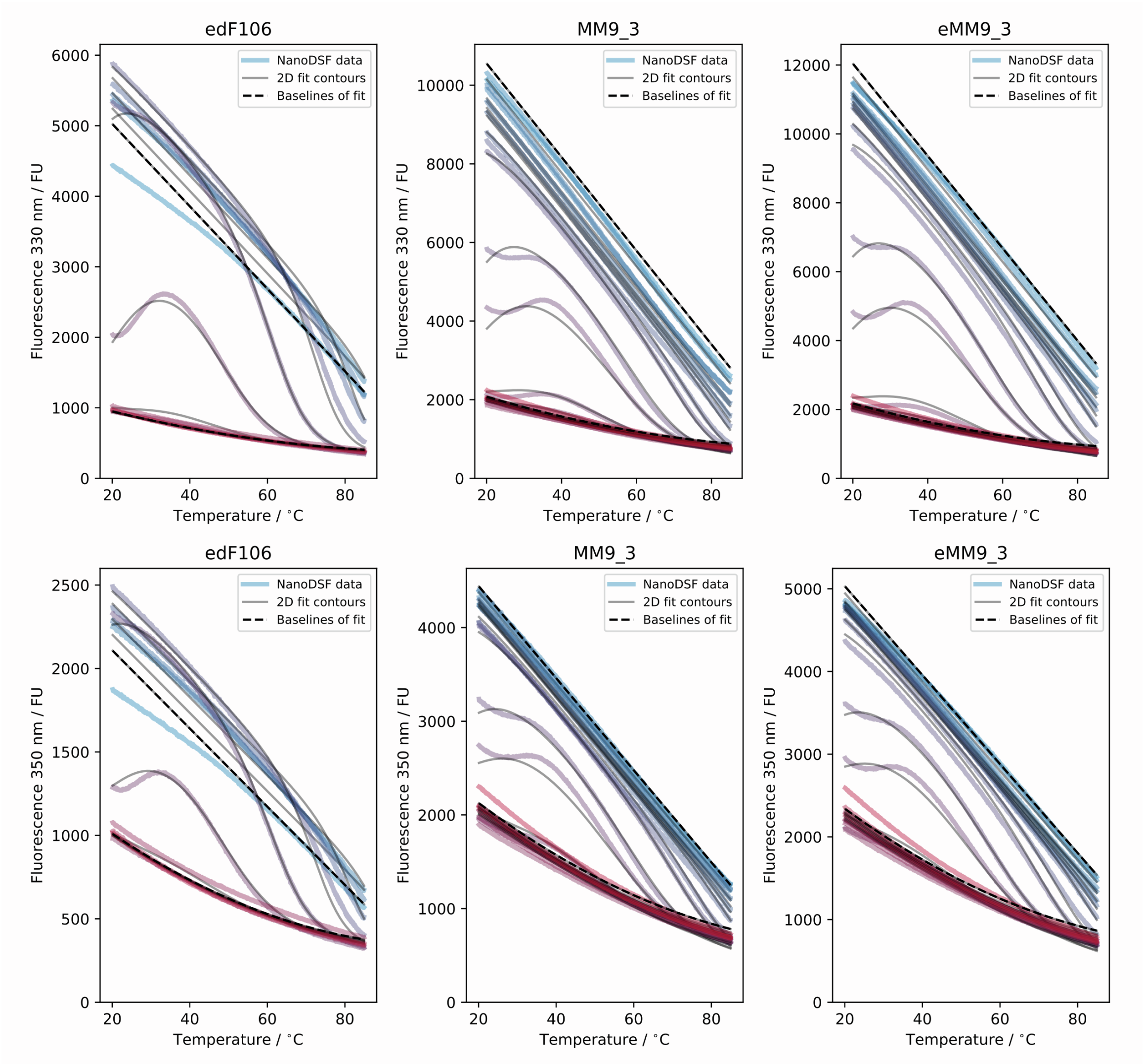

Figure S3: Fit evaluation for two-dimensional unfolding, Related to Figure 2. Full two-dimensional fits of edF106, MM9, and eMM9 are shown in the temperature dimension. The fluorescence intensities during unfolding at 330 nm and 350 nm when excited at 280 nm in NanoDSF are fitted. The samples are coloured from blue to red with increasing concentration of GuHCl: 0.6-6.1 M for edF106, 1.1-7.1 M for MM9, and 1.9-7.7 M for eMM9. Fitting was done globally on both traces of folding and the corresponding contours are shown in black with the highest and lowest concentration of GdnHCl shown as interrupted lines.

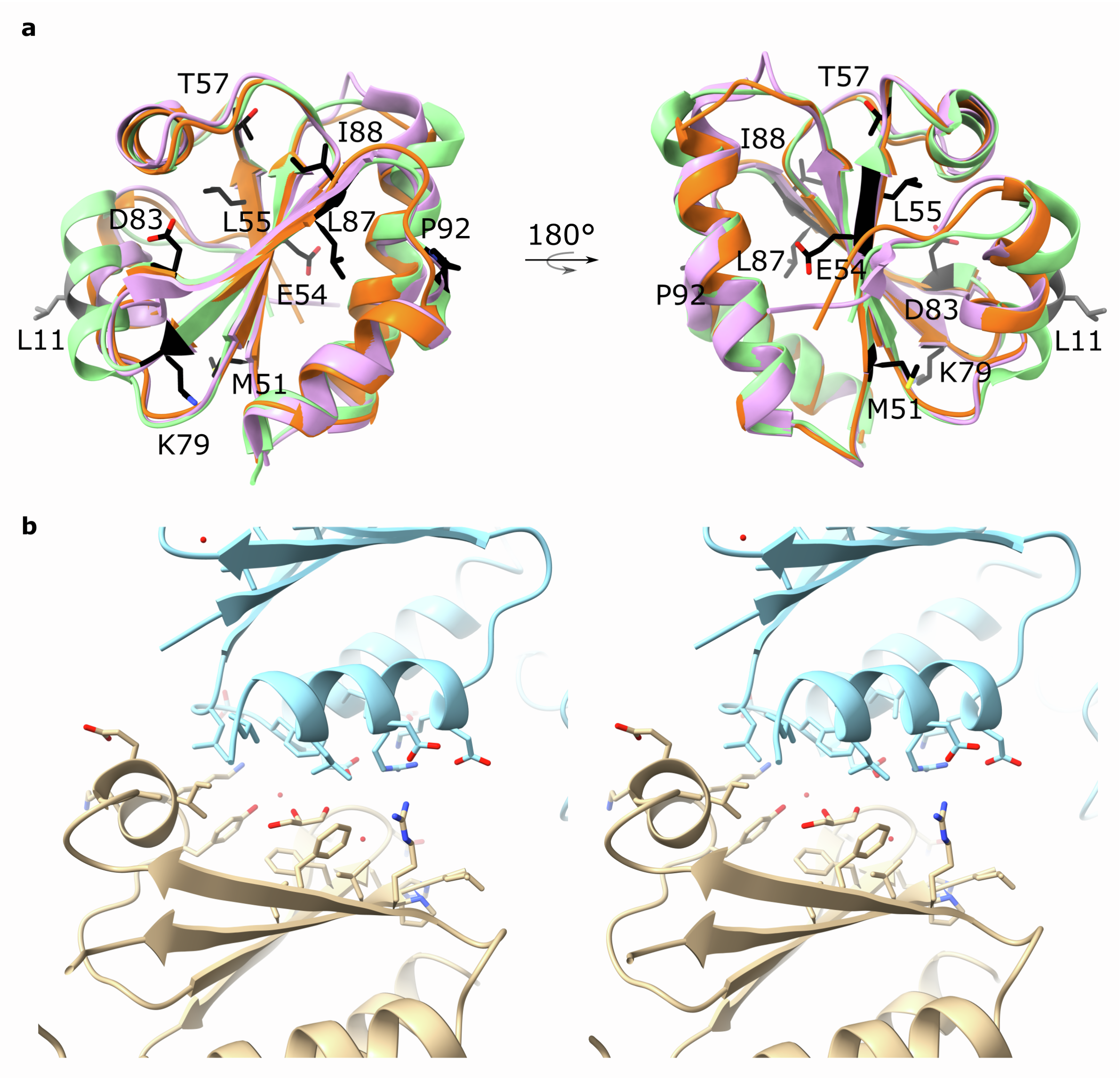

Figure S4: Structure changes maps poorly to substitutions, Related to Figure 3. a, Mutated residues to MM9 are drawn in stick representation and coloured in black. The residue L11 is coloured in grey because it was one of the original mutations in dF106 to make edF106. dF106 (PDB: 5J7D) in green, eMM9 (PDB: 7Q3K) in orange and the original spinach Trx design template (PDB: 1FB0) in purple. b, Stereo view of the crystal contact formed in the MM9 structure (PDB: 7Q3J) in the pocket normally occupied by the N-terminal. Here, the N-terminal has displaced and instead α-helix 4 and parts of α-helix 2 forms a symmetry contact involving a solvent glycerol molecule.
